# Levels, Distribution and Health Risk Assessment of Organochlorine Pesticides in Agricultural Soils from the Pearl River Delta of China

**DOI:** 10.3390/ijerph192013171

**Published:** 2022-10-13

**Authors:** Siyu Yao, Jiahui Huang, Haijun Zhou, Cuiting Cao, Tao Ai, Huanhuan Xing, Jianteng Sun

**Affiliations:** 1 Guangdong Provincial Key Laboratory of Petrochemical Pollution Processes and Control, School of Environmental Science and Engineering, Guangdong University of Petrochemical Technology, Maoming 525000, China; 2 Department of Environmental Sciences, College of Earth and Environment Sciences, Lanzhou University, Lanzhou 730000, China

**Keywords:** organochlorine pesticides, pearl river delta, agriculture soil, spatial distribution, source identification, human health risk

## Abstract

To reveal the pollution status of agricultural soils along with rapid urbanization and economic growth, a large regional survey of organochlorine pesticides (OCPs) in agricultural soils was conducted in the Pearl River Delta (PRD) of China. The results showed that the total residues of 23 OCPs were in the range of ND-946 ng/g dry weight. OCP residues showed distinct spatial distribution characteristics within the PRD. OCPs were mainly found in areas with high agricultural production and industrial activities. Higher OCP concentrations were observed in the top layer of soil, while the concentration decreases to marginal levels when the soil depth is greater than 50 cm. OCPs are mainly derived from historical use. Hexachlorocyclohexanes (HCHs) in the top soil of the study area are mainly from the use of lindane. Soil pH was negatively and significantly correlated with total OCP concentration. The human health risk assessment showed no health risk for children, while for adults, there is a non-carcinogenic risk, which needs to be noticed. Agricultural activities and industrial production have made the region a pollution hotspot and should arouse more stringent regulation to protect the environment and food safety.

## 1. Instruction

Organochlorine pesticides (OCPs) are a kind of chlorinated compound with high environmental persistence. In the middle of the 20th century, in order to prevent the invasion of pests and improve the harvest of crops, OCPs were widely used and promoted worldwide as a highly effective compound to compensate for the growing world population. However, due to the continuous overuse and abuse of OCPs, the environment and human health are now affected. Some OCPs are considered endocrine disrupting chemicals and systemic toxicants because of their ability to disrupt hormonal homeostasis and disrupt the endocrine system, thereby causing harm to humans and non-target organisms, such as acting as teratogens, disrupting neuroendocrine function, suppressing immunity and reproductive system function, and causing lipid and metabolic disorders [1,2,3].

According to the statistics of the World Health Organization, there are about three million cases of pesticide poisoning reported in the world every year, of which more than 10% lead to death [4,5]. Thus, OCPs are banned in most developed countries, but are still widely used in most third world countries because of their low price and availability. The use of persistent organic pollutants was not eased until the adoption of the Stockholm Convention in May 2001, and 12 pollutants were listed as persistent organic pollutants including nine OCPs [5].

Although most OCPs have been included in the Convention since 2001, due to the stability, refractory and lipophilicity of OCPs, they have caused a certain accumulation in the ecological environment and human beings [6,7,8]. Soil, as the primary accumulation medium for OCPs, is critical for the environmental cycling of these chemicals and has the potential to pose risks to human health and ecology [9]. Pokhrel [10] found high concentrations of OCPs in the soil of commercial land in Nepal. Zhang [11] detected OCP content by sampling the soil of vegetable fields in the suburbs of Changchun, Northeast China, and found that most of the organochlorine pesticides in soil samples originated from historical applications. Kafaei [12] found that most of the chlordane pesticide residues in agricultural soils in southern Iran came from historical applications, and they found that hexachlorocyclohexane (HCH) and dichlorotriphenyltrichloroethanes (DDTs) were recently used in the soils of the studied plains. Chen [13] found that OCPs also exist in the Tibetan Plateau; they found the local use of DDT, dicofol and HCHs may be an important source of the accumulation found in the soil of the Tibetan Plateau. In addition, aldrin and endosulfan are considered to be good indicators for studying atmospheric transport and deposition of OCPs from South Asia and Southeast Asia. Zhou [14] conducted a study on OCP contamination in breast milk from 12 provinces in mainland China. The results showed that DDTs were the most common drugs, followed by HCH and hexachlorobenzene (HCB).

For a long time, China’s population growth, rapid economic development, and other reasons, have resulted in the excessive use of chemical fertilizers and pesticides, and soil pesticide pollution has become a major environmental problem for the country. The soil pollution in the Pearl River Delta region is also becoming more and more serious due to the development of industry. The intensified agricultural and industrial activities may be one of the reasons for the increase in the level of various chemical pollutants in the environment. 

Twenty-three OCPs were classified into six major types for detection [15]. Thus, in this study, we classify organochlorine pesticides (OCPs) into six categories: HCHs (α-HCH, β-HCH, γ-HCH and δ-HCH); DDTs (p,p’-DDE, p,p’-DDT, o,p’-DDD, p,p’-DDD, o,p’-DDE and o,p’-DDT); DRINS (aldrin, dieldrin, mirex, Dieldrin); SULPHS (α-endosulfan, β-endosulfan); CHLS (cis-chlordane, trans-chlordane, cis-nonachlor, trans-nonachlor, epoxyheptachlor A and epoxyheptachlor B); and HCB (hexachlorobenzene). 

Through the analysis of 240 soil samples in the Pearl River Delta, we can investigate the current situation of OCP residues and provide basic data for the pollution by organic pollutants in the Pearl River Delta. 

## 2. Materials and Method

### 2.1. The Situation of the Pearl River Delta Region of China

The PRD is located in the south of the Tropic of Cancer, between 23°40′ and 21°30′ north latitude; that is, mostly in the tropical range with a subtropical climate. It is warm and humid all year round, has a rainy season and high temperature season synchronization, fertile soil, crisscrossing rivers, and is good for agriculture. The main crops in the PRD are rice, sugar cane, banana, and pineapple. It is the region with the fastest agricultural and rural economic development, and is an important vegetable, grain, and fruit production base in Guangdong Province. The soil environmental quality is closely related to people’s health.

### 2.2. Sample Collection and Preparation

A total of 240 topsoil samples were collected from various farmlands in the PRD. The sampling sites were located in Guangdong Province and samples were collected in the second half of 2019. At each sampling site, soil from five cores were collected using stainless steel and then composited into one single sample. In addition, 72 samples were collected for vertical soil profiles. The soil profiles were excavated to a depth of 80 cm. Soil samples were collected at 10 cm intervals. A total of 240 surface soil samples were collected (48 soil samples in Guangzhou, 30 soil samples in Shenzhen, 18 soil samples in Dongguan, 30 soil samples in Jiangmen, 16 soil samples in Zhongshan, 28 soil samples in Huizhou, 24 soil samples in Zhaoqing, 18 soil samples in Zhuhai, and 27 soil samples in Foshan), the latitude and longitude of the sampling points were recorded using GPS. 

The soil samples were stored at −20 ℃ until analysis. After freeze-drying, stones, plant tissues and other debris were removed from the samples, which were then ground and passed through a 1 mm sieve. The sample preparation and analysis procedures for OCPs were adapted from the reported methods [16]. A total of 6 g of soil sample was extracted with 20 mL of dichloromethane and hexane (1:1; *v/v*) in an ultrasonic bath for 30 min, and the extraction repeated 3 times. The extracts were concentrated and cleaned up by passing them through a Florisil column and eluted with 60 mL hexane and dichloromethane (4:1; *v/v*). Then, the elution was concentrated and exchanged into 1 mL hexane with a gentle nitrogen flow. Detailed information of the total organic carbon (TOC) is shown in Text S1. 

### 2.3. Sample Analysis

The quantification of OCPs was carried out using a GC MS-QP2010, Shimadzu, Japan with an HP-5MS quartz capillary column (30 m × 0.25 mm inner diameter ×0.25 μm film thickness). The ion source used by the mass spectrometer was an electron bombardment source (EI), and the ion monitoring (SIM) mode was used to quantitatively analyze the samples. The procedure is as follows: the initial temperature is 80 ℃ for 2 min, then it is heated to 140 ℃ for 10 min at a rate of 10 ℃/min, and then to 280 ℃ for 5 min at a rate of 4 ℃/min. 

### 2.4. Human Health Risk Assessment

The noncarcinogenic and carcinogenic risks of the OCPs were evaluated using the methods recommended by the USEPA [17]. A human health risk assessment of OCP exposure in urban farmland soil in the Pearl River Delta was conducted. Among the pollutants studied, α-HCH, γ-HCH and DDTs are considered to be non-carcinogenic compounds, while α-HCH, γ-HCH, β-HCH and DDTs are considered to be carcinogenic. Organic matter in soil enters the human body mainly via food ingestion, dermal contact and through the respiratory tract. Therefore, this study calculated and analyzed the non-carcinogenic risk and carcinogenic risk in different age groups via oral ingestion, skin contact and respiratory inhalation, so as to evaluate the health risk caused by OCP residues in urban farmland soil in the Pearl River Delta.

The average daily dose (ADD, mg·kg^−1^·day^−1^) of HCHs and DDTs via non-dietary (ingestion, dermal contact, and respiratory inhalation) pathways were calculated using the following equations:

$${\mathrm{ADD}}_{\mathrm{ingest}}=\frac{\mathrm{Cs}\times \mathrm{IR}\times \mathrm{CF}\times \mathrm{EF}\times \mathrm{ED}}{\mathrm{BW}\times \mathrm{AT}}\times \mathrm{CF}\;{\mathrm{ADD}}_{\mathrm{dermal}}=\frac{\mathrm{Cs}\times \mathrm{CF}\times \mathrm{SA}\times \mathrm{AF}\times \mathrm{ABS}\times \mathrm{EF}\times \mathrm{ED}}{\mathrm{BW}\times \mathrm{AT}}\times \mathrm{CF}\;{\mathrm{ADD}}_{\mathrm{inhale}}=\frac{\mathrm{Cs}\times \mathrm{IhR}\times \mathrm{EF}\times \mathrm{ED}}{\mathrm{PEF}\times \mathrm{BW}\times \mathrm{AT}}\times \mathrm{CF}$$

A hazard quotient (*HQ*) was introduced to estimate the non-cancer risks of HCHs and DDTs in soils via non-dietary pathways. It is based on the *ADD* and the specific reference dose (*RfD*) with the following equations:

$$HQ=\frac{ADD}{RfD}\;HI=\sum HQi=\sum \frac{ADDi}{RfDi}$$

The cancer risk assessments on humans were estimated using the ADD and multiplying by a slope factor (SF). The commonly used expression of risk value R of carcinogenic risk is:

R=ADD×SF

The representative meanings and reference values of each parameter in the above formula are shown in Table S1. The RfD and SF values required for evaluation in this paper mainly refer to the integrated Risk Information System (IRIS, USA) database, and the specific reference values are shown in Table S2.

### 2.5. Quality Assurance and Quality Control

Quality controls were implemented to ensure the correct identification and accurate quantification of the target analyses. A method-blank sample was included in every batch of 15 samples to control any systematic contamination. The recovery rates of 23 OCPs in the samples ranged from 75.2 to 107.8%. The RSD for replicate analyses was lower than 15% (n = 3). During routine sample analysis, a duplicate was included in every batch of 15 samples with an RSD of detected concentration lower than 10% (n = 3).

### 2.6. Data Processing and Statistical Analysis

Statistical analyses were performed using Microsoft Excel 2016, Origin 10.6 (OriginLab Inc., Northampton, MA, USA) and SPSS 22.0 (IBM, Armonk, NY, USA). A Spearman correlation analysis was used to evaluate the relationships between the contaminant concentrations in soil, total organic carbon (TOC) in soil, and the pH. ArcGIS 10.5 was used to map the spatial distribution of 23 OCPs in soil in the Pearl River Delta.

## 3. Results and Discussion

### 3.1. Residual Status of OCPs in the Pearl River Delta

The status of OCP residues in the Pearl River Delta is shown in Table 1. The total concentration of residual OCPs in the 240 soil samples in the study area ranged from ND to 946 ng/g dry weight, with an average concentration of 65.5 ng/g and a detection rate of 89.6%. Among them, DDTs accounted for the largest share of the total OCPs, with concentrations ranging from ND to 743 ng/g, and the average value was 34.2 ng/g; followed by HCHs, with concentrations ranging from ND to 110 ng/g, and an average value of 4.52 ng/g. Thus, it can be seen that DDTs and HCHs are the main components of OCP pollution in the Pearl River Delta. In addition to DDTs and HCHs, other important OCPs, including DRINs, SULPHs, CHLs and HCB, were also found in this study, reflecting the pesticide use habits in local agricultural production activities. The detection rates of 23 OCPs were higher than 50% only for DDTs (65.4%) and HCHs (64.2%), while the detection rates of the rest of the contaminants did not exceed 50%; therefore, the distribution of DDTs and HCHs in the soil were mainly studied in this study.

The overall coefficient of variation of OCPs was as high as 216%, indicating that there is considerable dispersion in the use of organochlorine pesticides in the PRD region, with a large variation in the content of OCPs from region to region and a high degree of their local enrichment, which generally reflects the disorderly use of pesticides, mostly in a fragmented production and management mode [15], and more obviously influenced by human activities.

According to China’s Soil Environmental Quality Standard (GB-156182018) (China State Environmental Protection Administration, 2018), the risk screening limits of HCHs and DDTs in agricultural soils are 100 ng/g, respectively, and the rest of the organic pesticides under this amount are not counted due to a lack of information. According to the results of this study, the concentrations of HCHs and DDTs in 240 agricultural soils in the Pearl River Delta exceeded this safety standard in 1 and 17 soil samples, with exceedance rates of 0.42% and 7.08%, respectively, both of which were higher than those in the Yangtze River Delta and the Pearl River Delta in 2008 (Yangtze River Delta: the exceedance rate of HCHs was 0 and DDTs was 6.6%; 2008 Pearl River Delta: the exceedance rate of HCHs was 0 and the exceedance rate of DDTs was 2.3%) [16,18]. The residue levels of OCPs in this study were comparable to those in the Yangtze River Delta (DDTs: mean concentration 56.2 ng/g; HCHs: mean concentration 2.46 ng/g; total mean concentration: 59.3 ng/g) [16] and are applicable to similar industrial and agricultural activities. Compared with 2008, the residues of OCPs in agricultural soils in the Pearl River Delta (DDTs: average concentration 12.3 ng/g; HCHs: average concentration 1.87 ng/g; total average concentration: 20.67 ng/g) [18] showed a significant increasing trend.

The increase in OCPs must be related to the use of organic pesticides in the Pearl River Delta in the last decade. Meanwhile, the diversity of latitude and longitude makes the difference in temperature, environment and soil organic matter also lead to differences in the residual concentrations of OCPs in different cities (Table 2).

The PRD region is located at the southern end of the subtropics, with sufficient light and heat, abundant rainfall and water resources, a high degree of soil weathering and leaching, and high activity of microorganisms, thus accelerating the degradation rate of HCHs and DDTs. In addition, the soils were often washed and drenched by rainwater, which can accelerate the migration and transformation rate of HCHs and DDTs in agricultural soils in the PRD. In China, the use of HCHs is higher than that of DDTs, but in the soil, DDTs and its degradation products are in higher concentration than HCHs because DDTs have high stability and degrade more slowly than HCHs.

### 3.2. Spatial Distribution Characteristics of OCPs in the Pearl River Delta

**Figure 1:**
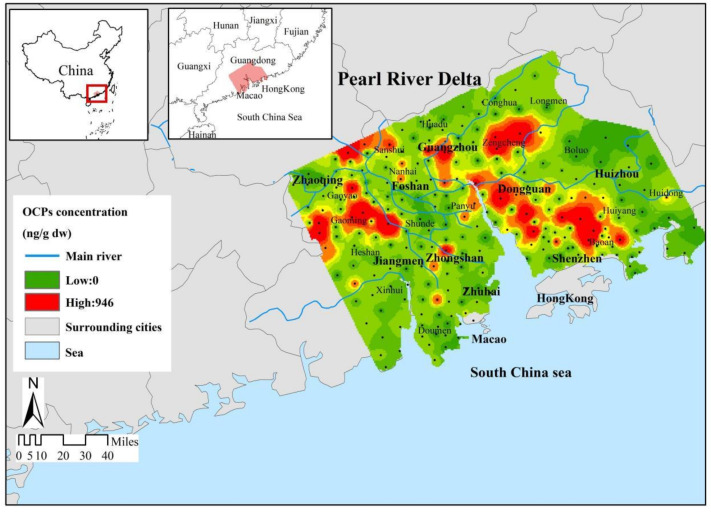
Spatial distribution of residual OCP concentration in agricultural soil in the PRD.

The spatial distribution of the concentration of sampling sites were drawn based on the residual concentration of OCPs in the surface soil, as presented in Figure 1 and Figure S1. The regional characteristics of the distribution of OCPs in the PRD are relative obviously, and higher concentration of OCPs were found in cities with high population density and frequent agricultural activities, such as Dongguan and Zhaoqing, indicating that agricultural activities related to pest control needs may cause OCP pollution [33]. The same higher concentration of OCPs were found in cities where industrial production is intensive, such as in Shenzhen, Foshan, and Guangzhou, indicating that a large number of factories may be another reason for OCP pollution.

While the transition region mainly evolved into intermediate values, the low value areas were mainly distributed in the southeast of the PRD. The residual concentrations of soil OCPs in the study area belong to the middle and lower levels, and the areas with a high content of total OCPs (>200 ng/g) are mainly concentrated in Dongguan, Guangzhou, Shenzhen, and Foshan. The intermediate values (50~200 ng/g) were mainly found in the plains of the PRD, covering about 65% of the whole region; the low content areas (<50 ng/g) were mainly found in the fringes of the east and west of the PRD, mainly in the mountains and hills. By combining Table 1 and Figure 1, it can be seen that the highest value of OCPs appeared in Foshan, which was 946 ng/g.

### 3.3. Vertical Distribution Characteristics of OCP in Soil Profiles

**Figure 2:**
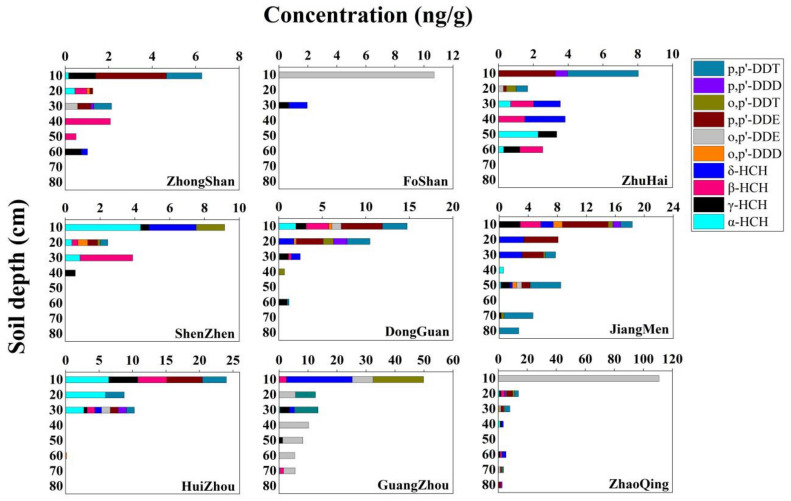
Vertical distributions of the concentrations and compositions of OCPs in agricultural soils of the PRD.

The vertical concentration and distribution of OCPs in soil at different depths in the cities of the PRD were mapped and the results are shown in Figure 2. The OCP residues varied from cities to cities, which may be related to local agricultural activity habits and industry, such as in Guangzhou and Zhaoqing. However, in the vertical soils of all cities, it is evident that higher OCP concentrations can be observed in the top layer of soil distributed between 0–50 cm, while the concentration of OCPs decreases to marginal levels when the soil depth is greater than 50 cm. OCPs are classes of chemically stable and difficult to decompose organic substances, and most OCPs are hydrophobic, lipid-soluble compounds, resulting in the impossibility of losing a large amount of OCPs in the soil via their infiltration to the subsurface layer, but are, instead, more adsorbed by soil particles. Therefore, in the soil profile, higher concentrations of OCPs are only found in the upper layer of soils with more agricultural and industrial activities. The concentration of OCPs decreases with soil depth, indicating that OCPs do not cause secondary pollution through groundwater infiltration. This result is similar to that of Sun [16].

### 3.4. Source Identification of OCPs in the Pearl River Delta

#### 3.4.1. HCHs

There are two types of organochlorine pesticides used commercially, industrial HCH and lindane. Industrial HCHs mainly contain 60% to 70% α-HCH, 5% to 12% β-HCH, 10% to 12% γ-HCH, 6% to 10% δ-HCH and others; while in lindane, the main component consists of γ-HCH (99%) [34]. The results of the determination of HCHs from all samples in the PRD region are shown in Table 1, from which it can be seen that the residual levels of HCHs in the study area ranged from ND to 110 ng/g, with the detection rates of α-, β-, γ- and δ-HCH being 32.5%, 31.3%, 35.8%, and 32.1%, respectively. Among the HCHs, microorganisms in soil do not easily degrade β-HCH either, because of its lower saturated vapor pressure, high melting and boiling point, and higher bioaccumulation properties than other isomers. α-HCH and γ-HCH in soil will be converted to β-HCH, which is relatively stable in nature, via long time residues, if no other HCHs are inputted [35]. The possible interconversion between the isomers makes the composition characteristics of each isomer in HCH residues in the environment able to be used as an environmental indicator. Therefore, a number of relevant studies have indicated that the α-HCH/γ-HCH ratio can be used to distinguish the main sources of HCHs in soil [18,36]. In general, when α-HCH/y-HCH in soil is < 3, it indicates that HCHs in the area mainly originate from the input of lindane; when α-HCH/γ-HCH is > 7, it indicates that they probably originated from industrial HCH use and the contaminants have been degraded over a long period of time. In this study, we first calculate the ratios of each sampling site, then average the ratios to obtain the ratios of isomers of HCHs and DDTs. The α-HCH/γ-HCH ratios in the surface soil samples of the study area were generally lower than three, indicating that the HCHs in the soil at most of the sampling sites were likely to be sourced from lindane.

To analyze whether HCHs in the PRD are historical residues or recent inputs, the results of β-HCH/(α-HCH+γ-HCH) was used in this study to make a judgment [37]. When β-HCH/(α-HCH+γ-HCH) is higher than 0.5, it indicates that the source of the HCHs is mainly historical residues, and when the ratio is lower than 0.5, the HCHs in the soil are considered to be mainly recent new inputs of contaminants [38]. In this study, the mean value of β-HCH/(α-HCH+γ-HCH) in all 240 samples was 3.72, indicating that the residues of the HCHs in this study area were mainly historical residues. In conclusion, the source of the HCHs in the top soil of the study area is mainly due to the use of lindane and historical application residual contamination.

#### 3.4.2. DDTs

From Table 1, the total detection rate of DDT isomers and metabolites (o,p’-DDT, p,p’-DDT, o,p’-DDD, p,p’-DDD, o,p’-DDE and p,p’-DDE) was 65.4%, and the residue levels ranged from ND to 743 ng/g with a mean value of 34.2 ng/g. The average level of DDT residues was higher than that of HCHs, with the highest detection rate of p,p’-DDE and the highest residue concentration of p,p’-DDT.

Initially, industrial DDT was used in the environment, mainly consisting of 75% p,p’-DDT, 15% o,p’-DDT, 5% p,p’-DDD, 5% p,p’-DDE and other substances [39]. However, some studies have shown that after the ban of industrial DDT, dicofol is slowly becoming a new source input of DDT in the environment. Dicofol and industrial DDTs differ in composition, with dicofol being rich in o,p’-DDT, with a ratio of o,p’-DDT/p,p’-DDT above 1.3 to 9.3, while industrial DDT has an o,p’-DDT/p,p’-DDT of only 0.2 to 0.3. Therefore, the ratio of o,p’-DDT/p,p’-DDT can be used to discriminate the presence or absence of dicofol input in the soil environment. From the composition of DDT isomers in PRD soils, the o,p’-DDT/p,p’-DDT ratios are all below 0.3, indicating that the soils in the region are dominated by industrial sources of DDTs [18].

In the soil environment, DDT is degraded in two ways, aerobic decomposition and anaerobic decomposition, under the action of microorganisms. DDT reacts under aerobic conditions to form DDE, and in anaerobic environment to form DDD, so the magnitude of (DDE+DDD)/DDT can be used to indicate the degradation of DDT and to determine whether the contamination of DDTs is a historical residue or a new input [40]. If (DDE+DDD)/DDT is > 1, it means that DDTs are mainly from historical residues and degradation is relatively complete; on the contrary, if (DDE+DDD)/DDT is < 1, this indicates that the pollutants are mainly from new inputs [41]. The mean value of (DDE+DDD)/DDT in the surface soil of the Pearl River Delta was 1.43, indicating that the DDTs were mainly from historical residues. In conclusion, it can be concluded that historical residues of industrial DDTs are the main source of DDTs in agricultural soils in the PRD.

#### 3.4.3. Other OCPs

The main components of industrial endosulfan are α-endosulfan and β-endosulfan in a 7:3 ratio. α-endosulfan is more volatile and degradable than β-endosulfan in soils [42,43]. In this study, α-endosulfan had the highest detection rate of 22.6% with a mean value of 13.0 ng/g. The mean concentration of α-endosulfan in soil did not differ much from most cities in other countries, except for West Ethiopia [44] and Goa, India [45]. Although β-endosulfan was generally not detected in this study, the results based on α-endosulfan/β-endosulfan suggest that there is no new input of endosulfan in the region, which mainly originates from historical use and may have been degraded to endosulfan sulfate.

Industrial chlordane is the main source of trans and cis chlordane in the soil environment, and its main components include 11% cis chlordane, 13% trans chlordane, 5% heptachlor, and 5% trans heptachlor [46]. In the soil environment, trans-chlordane is degraded at a faster rate than cis-chlordane, so the size of trans-chlordane/cis-chlordane is often used to determine the residual status of chlordane in soil [47]. The ratio of industrial chlordane is about 1.18, and when trans/cis chlordane > 1.18, it indicates a recent input of chlordane to the study area, and conversely, if trans/cis chlordane < 1, it indicates that chlordane in the soil originated from historical use. In the present study, the ratio was generally below 1.18, indicating that industrial chlordane in the surface soils of the region was derived from residues of historical use.

### 3.5. Correlations among Soil Properties and Pollutants

Soil is a system with a rich composition, mainly consisting of solid, liquid and gas phase systems, and the components of the soil system can be broadly divided into soil organisms, soil minerals, soil organic matter, liquid, and air. The migration of organochlorine pesticides in the soil is generally achieved by diffusion, water infiltration and other effects. Therefore, the distribution characteristics and migration patterns of organochlorine pesticides in soil are not only determined by the nature of the contaminants themselves [48], but also may be related to soil physicochemical properties such as pH, temperature, moisture, porosity, and organic matter content in the soil. In this study, the correlation between soil physicochemical properties and the distribution characteristics of organochlorine pesticides in various regions of the PRD was mainly analyzed and determined (Table S3). The correlation between OCP concentration and soil properties is shown in Figure S2.

pH values in the PRD region ranged from 5.00 to 8.87, with an average pH of 6.69, the soil was acidic in a few areas and weakly alkaline in most areas. A Spearman correlation analysis showed that the pH was negatively correlated with the concentration of total soil OCPs (r = −0.191, *p* < 0.01), which was similar to the findings of Gong, Gao, and Zhang [23,48,49]. This is probably because soil pH has an effect on the composition and structure of humic acid. Some studies have shown that humic acid particles become smaller with increasing pH, while increasing pH decreases the number of voids in humic acid polymers and reduces the number of interaction sites [50]. In the present study, soil pH was negatively correlated with total OCP concentration, and it is speculated that the reason for this may be that changes in pH have some effect on the composition and structure of humic acids in the soil, which in turn leads to changes in soil OCP concentrations.

As hydrophobic substances, OCPs are more inclined to be adsorbed by soil organic matter, and the high concentration of organic matter can provide a sufficient carbon source for microorganisms in soil, thus accelerating the decomposition of OCPs by microorganisms. In this study, the TOC in various areas of the Pearl River Delta ranged from 0.08–46.4 g/kg with a mean value of 12.9 g/kg. The Spearman correlation analysis showed that there was no significant correlation between TOC and total OCP concentrations. This result is similar to the results of Sun [51], who concluded that there was no significant correlation between OCPs and TOC in the field soil by comparing the correlation between OCP concentrations and TOC in plastic greenhouses and field soil in northern China. Although there are many reports showing that OCPs are significantly correlated with TOC, the results cannot be generalized, and different conditions may affect the relationship between OCPs and TOC. It has been shown that in an air-soil system in equilibrium, there is a proportional relationship between the soil organic matter content and the concentration of hydrophobic compounds in soil [52,53]. The sampling areas in the Pearl River Delta are all open land rather than closed space, and the equilibrium state is relatively difficult to reach. Therefore, the lack of significant correlation between OCPs and TOC may be due to the distribution of OCPs in the soil and the adsorption state of TOC in the soil did not reach equilibrium. In conclusion, environmental factors, such as pH, can affect the residual levels of OCPs in soil to some extent, but the specific mechanism of the effect of TOC on soil OCPs residues needs further study.

### 3.6. Human Health Risk Assessment

#### 3.6.1. Non-Carcinogenic Health Risk Evaluation

**Figure 3:**
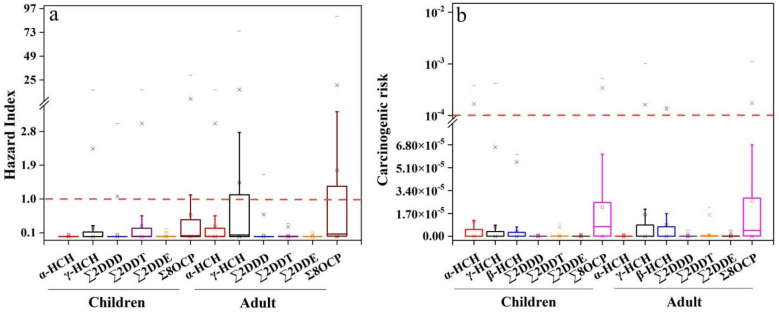
(**a**) Non-carcinogenic risks and (**b**) carcinogenic risks to children and adults through dietary and non-dietary exposure pathways to OCPs.

The U.S. National Environmental Protection Agency (USEPA) stipulates that for cumulative non-carcinogenic risk, if the cumulative non-carcinogenic risk value of OCPs is HI < 1, the non-carcinogenic risk is relatively small or negligible. On the contrary, if the cumulative non-carcinogenic risk value of OCPs is HI > 1, it will have harmful effects on human health and there is non-carcinogenic risk. The study evaluated the non-carcinogenic risk of OCPs in urban agricultural soils in the PRD for different age stages and different exposure routes, and the evaluation results are shown in Figure 3a. For the average non-carcinogenic risk of OCPs (HI = 0.57 for children and HI = 1.76 for adults), the evaluation results showed that the OCPs had no non-carcinogenic risk for children, while the OCPs had a non-carcinogenic risk for adults. The non-carcinogenic risks of o,p’-DDT, p,p’-DDT and γ-HCH were relatively large. The non-carcinogenic risk values of PRD soils were lower than the USEPA in the children group (HI < 1), but the mean non-carcinogenic risk in the adult group was > 1, indicating that OCPs in PRD urban agricultural soils have no non-carcinogenic risk to children’s bodies, but have some non-carcinogenic risk to adults and is of concern.

#### 3.6.2. Carcinogenic Health Risk Evaluation

For different age stages and different exposure routes, the carcinogenic risk of OCPs in the soil of urban agricultural fields in the PRD was calculated and analyzed, in which the non-carcinogenic health risk values caused by oral intake, respiratory intake and dermal contact are shown in Figure 3b, respectively. It is generally accepted that the carcinogenic risk is very high when the carcinogenic risk is > 10^−1^, high when 10^−3^ < carcinogenic risk < 10^−1^, moderate when 10^−4^ < carcinogenic risk < 10^−3^, low when 10^−6^ < carcinogenic risk < 10^−4^, and very low when carcinogenic risk < 10^−6^. In this study, the carcinogenic risk values of OCPs in soil at most sites were small and the risk level was very low or low. As shown in Figure 3b, γ-HCH and β-HCH were the main contributors to the carcinogenic risk of OCPs. Overall, HCHs and DDTs in the soil of the PRD urban farmland pose, essentially, no carcinogenic risk to the health of local residents, whether through oral or dermal exposure or through inhalation.

## 4. Conclusions

This study reveals the current status of OCPs in agricultural soils in the PRD region. The residuals of OCPs show quite different spatial distributions. Higher concentrations of OCPs were found in cities with high agricultural activities, such as Zhaoqing and Dongguan, and in cities with intensive industrial production activities, such as Guangzhou and Shenzhen, where the rapid development of agriculture and industry is considered to be the main source of OCPs.

Soil profiles showed that OCP concentrations decreased to marginal levels in soils greater than 50 cm in depth, indicating that OCPs do not cause contamination through groundwater infiltration. The historical use of industrial DDT was considered the main source of DDTs, while the source of HCHs was mainly due to lindane use and residual contamination from historical use. The concentrations of OCPs were negatively correlated with pH and not significantly correlated with TOC. Based on the assessment of this study, the health risk from OCP contamination in soil is generally low for children, but there is still some non-carcinogenic risk for adults.

Although OCPs are now listed in the Stockholm Convention as chemicals to control, there are regions where OCPs are still being used because they are inexpensive and readily available. Therefore, future research should focus on reducing the use of OCPs. On this basis, to reduce the accumulation of OCPs in agricultural soils, we propose the development and use of biochar to decrease the accessibility of OCPs, the enhancement of soil microbial communities, and that the studied area should also be continuously monitored [54].

## Figures and Tables

**Table 1:** Concentration of 23 OCPs in agricultural soil samples of the Pearl River Delta.

Compound	Min ng/g	Max ng/g	Mean ng/g	Detection Rate /%
α-HCH	ND ^a^	37.7	1.09	32.5
γ-HCH	ND	89.0	1.40	35.8
β-HCH	ND	11.8	0.70	31.3
δ-HCH	ND	68.3	1.33	32.1
ΣHCHs	ND	110	4.52	64.2
o,p’-DDD	ND	165	2.33	15.0
o,p’-DDE	ND	174	3.80	23.8
p,p’-DDE	ND	97.6	2.06	28.3
o,p’-DDT	ND	489	15.2	27.9
p,p’-DDD	ND	58.6	0.34	5.42
p,p’-DDT	ND	580	10.5	27.5
ΣDDTs	ND	743	34.2	65.4
Aldrin	ND	5.80	0.04	0.83
Dieldrin	ND	12.6	0.17	2.50
Endrin	ND	283	2.81	3.33
Mirex	ND	29.2	0.51	5.42
α-Endosulfan	ND	288	13.0	22.5
β-Endosulfan	ND	ND	-	-
cis-chlordane	ND	20.4	0.63	9.17
Trans-Chlordane	ND	8.70	0.08	1.25
heptachlor epoxide isomer A	ND	113	3.89	21.3
heptachlor epoxide isomer B	ND	102	0.43	0.42
Cis-Nonachlor	ND	90.4	0.63	2.92
trans-Nonachlor	ND	ND	-	-
HCB	ND	41.5	0.40	4.58
ΣOCPs	ND	946	65.5	89.6

^a^ ND, none-detectable.

**Table 2:** Residual concentrations of HCHs and DDTs in soils of different regions.

Regions	Year of Sampling	HCHs ng/g (Mean)	DDTs ng/g (Mean)	References
Beijing	2003	0.64~32.32 (1.47)	1.42~5910.80 (77.18)	[19]
Nanjing	2002–2003	2.7~130.6 (13.6)	6.3~1050.7 (64.1)	[20]
Hong Kong	2002–2003	2.5~11 (6.19)	ND~5.7 (0.52)	[21]
Guangzhou	1999, 2002	0.19~42.3 (4.39)	3.58~831 (81.4)	[22]
Tianjin	2001	1.3~1094.6 (45.8)	0.071~972.24 (56.01)	[23]
Yangtze River Delta	2014	0.37~30.3 (2.46)	0.13~3515 (56.2)	[16]
Pearl River Delta	2008	ND~41.62 (1.87)	ND~383.41 (12.30)	[18]
Ningbo	——	2.7~28.2 (4.6)	2.2~566.6 (55.6)	[24]
Southern Italy	2015	ND~0.72 (0.065)	ND~16.4 (1.22)	[25]
Southern Nigeria	——	ND~156 (15.5)	——	[26]
Korea	2017	ND~14.51 (0.2)	ND~2187.18 (23.18)	[27]
Tanzania	2019	ND~1.54 (0.234)	ND~47.44 (2.29)	[28]
Campanian Plain	2011	0.03~17.3 (1.38)	0.08~1231 (107)	[29]
Dibrugarh	2009-2010	178~1701 (705)	75~2296 (757)	[30]
Midway Atoll	2006	ND~127 (23.5)	1.4~643 (191)	[31]
Galicia, NW Spain	2003	ND~2305 (200)	——	[32]
Pearl River Delta	2019	ND~110 (4.52)	ND~743 (34.2)	This study

## Data Availability

Not applicable.

## Supplementary Materials

The following supporting information can be downloaded at: https://www.mdpi.com/article/10.3390/ijerph192013171/s1, Figure S1: Concentration of OCPs of different cities; Figure S2: The relationship between OCPs and environmental factors (pH, TOC), Linear regressions were used to test the Spearman correlation between OCPs and pH and TOC; Table S1: Parameters used in exposure cancer and no-cancer risk assessments; Table S2: Parameters used in exposure cancer and no-cancer risk assessments in different pathways; Table S3: Physical Chemical Properties of Soils; Text S1: Soil physicochemical test.
