# Supplementary material for: Levels, Distribution and Health Risk Assessment of Organochlorine Pesticides in Agricultural Soils from the Pearl River Delta of China
Source: Int J Environ Res Public Health. 2022 Oct 13;19(20):13171. doi: 10.3390/ijerph192013171 (PMC9603595; doi:10.3390/ijerph192013171)
Supplement: Supplementary file 1 [file ijerph-19-13171-s001.zip › ijerph-1882760-supplementary.pdf]

International Journal of
Environmental Research and Public Health

MDPI

Supplementary Material

Text S1 Soil physicochemical test

Used dilution heat method for TOC analysis. Weigh 0.5000 g of soil sample accurately in a 500 mL triangular flask and add 10 mL of 1 mol/L (1/6 K2Cr2O7) solution accurately to it. Shake the flask gently to mix evenly. Subsequently, 20mL of concentrated sulphuric acid was slowly added and slowly rotated for 1min to allow sufficient mixing action with the soil sample. The triangular flask was placed on an asbestos sheet for 30 min, diluted to 250 mL with water and titrated with 3-4 drops of ophthalmic rolene indicator, using 0.5 mol/L FeSO4 standard solution with a brick red end colour.

The calculation formula are as followed:

TOC（g/Kg）=

$$\frac{c\ (V0 - V)\ * 10 - 3 * 3.0 * 1.33}{Drying\ soil\ weight} * 1000$$

Soil organic matter= TOC*1.724

1.33——Oxidation correction factor

C——Concentration of 0.5mol/L$FeSO_4$ Standard solutions

Table S1. Parameters used in exposure cancer and no-cancer risk assessments.

Parameter		Children	Adult	representative meaning
IR	mg·day^{-1}	200	100	Soil ingestion rate
SA	cm^2	2373	6032	Soil surface area
AF	mg·cm^{-2}	0.2	0.07	Soil adherence factor
CF	kg·mg^{-1}	1×10^{-6}	1×10^{-6}	Conversion factor
EF	day·yr^{-1}	350	350	Exposure frequency
ED	yr	6	26	Exposure duration
PEF	m^3•kg^{-1}	1.4×10^9	1.4×10^9	Particulate emission factor
IhR	m^3•day^{-1}	12	13.25	Inhalation rate
BW	Kg	15	80	Body weight
ABS	-	0.1	0.1	Fraction of contaminant absorbed dermally from soil
AT	day	2190/25550	9490/25550	Average lifetime

Table S2. Parameters used in exposure cancer and no-cancer risk assessments in different pathways.

	RfD	SF（kg•d/mg）
Ingest	2 ×10^{-5}	2
Dermal	2 ×10^{-5}	2
Inhale	2 ×10^{-5}	2.18 ×10^{-3}

Table S3. physical Chemical Properties of Soils.

	pH	TOC（g/kg）
Max	8.87	46.4
Min	5.00	0.08
Average	6.69	12.9

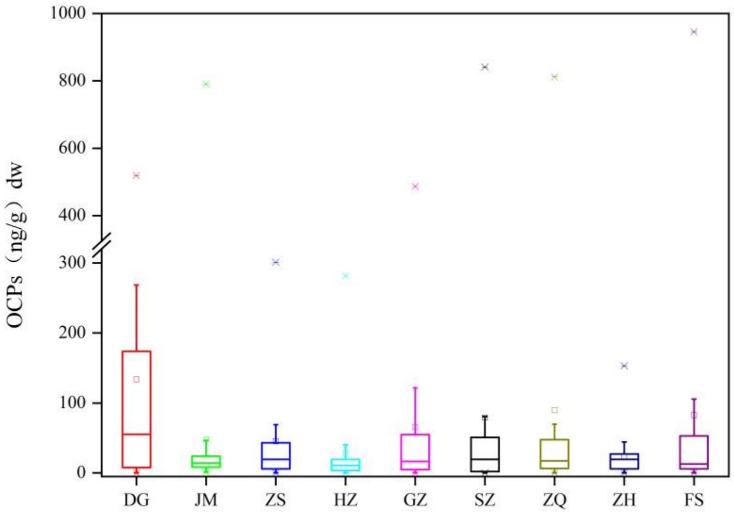

Figure S1. Concentration of OCPs of different cities.

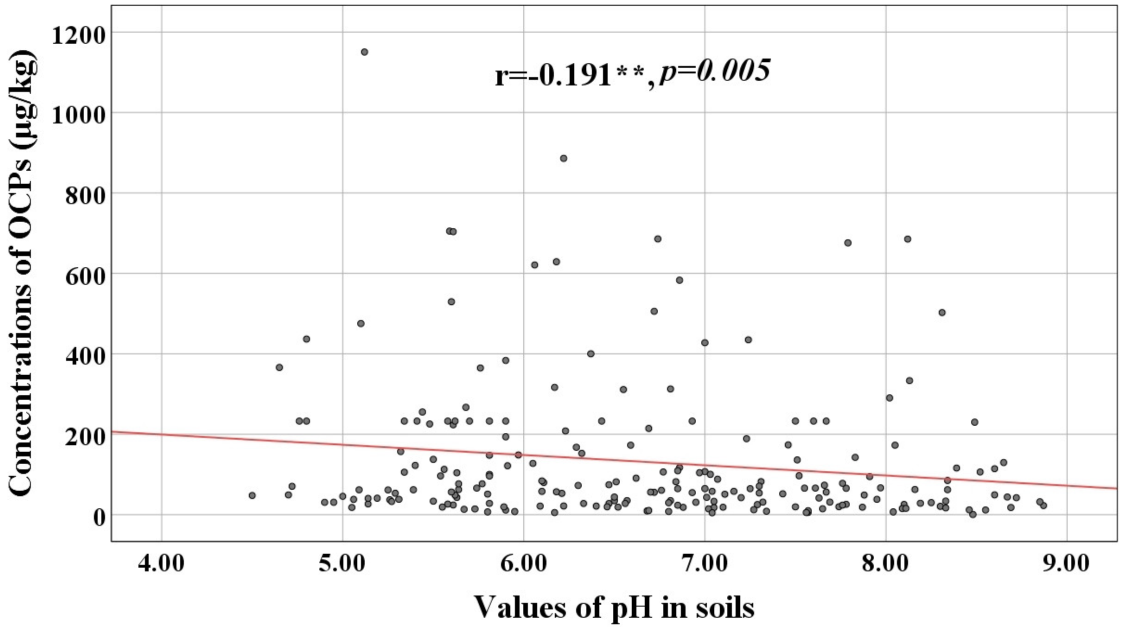

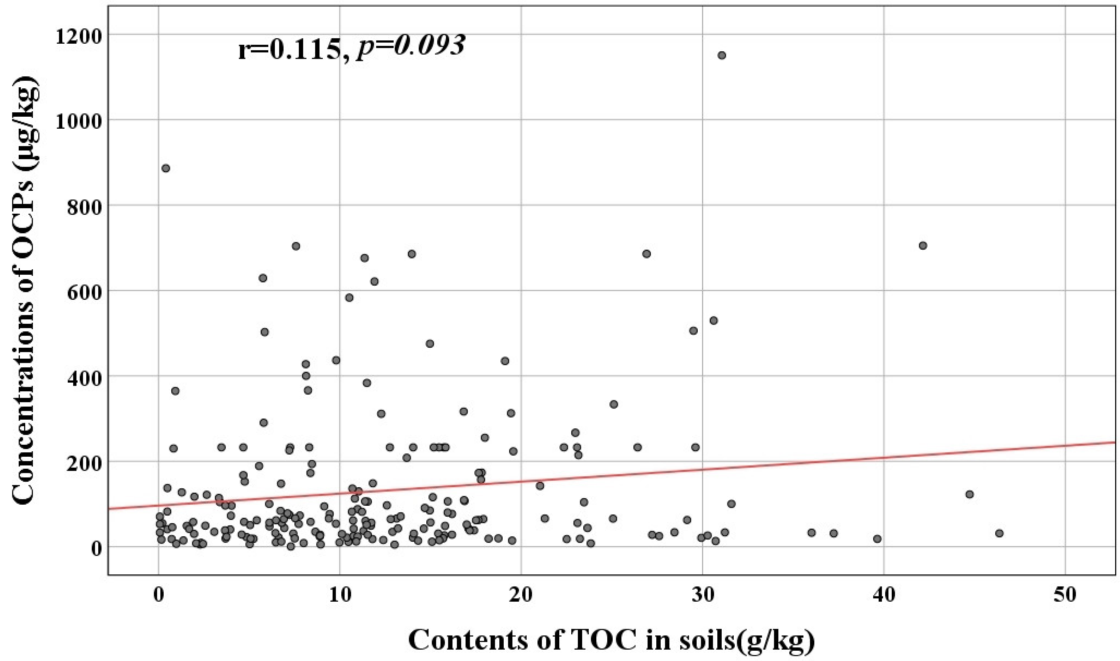

Figure S2. The relationship between OCPs and environmental factors (pH、TOC), Linear regressions were used to test the Spearman correlation between OCPs and pH and TOC.
